# Variability in United States online rehabilitation protocols after open and endoscopic hip abductor repair

**DOI:** 10.1007/s00402-026-06451-9

**Published:** 2026-08-03

**Authors:** Chukwudubem Anwunah, Samuel Rosenberg, Matthew Hartwell

**Affiliations:** University of Michigan-Ann Arbor, Ann Arbor, USA

**Keywords:** Hip abductor repair, Postoperative rehabilitation, Physical therapy, Standardization, Protocol variability

## Abstract

**Purpose:**

The purpose of this study was to evaluate readily accessible U.S. based online physical therapy protocols for open and endoscopic hip abductor repairs and to assess the variability in post-operative rehabilitation protocols.

**Methods:**

Online physical therapy protocols from U.S. based orthopaedic institutions for gluteus medius/minimus repair were collected using a web-based query utilizing search terms “hip abductor repair rehabilitation protocol” and “gluteus repair rehabilitation protocol.” Published protocols from both Electronic Residency Application Service (ERAS) academic orthopaedic surgery programs and private groups were collected. All publicly available protocols were included. Protocols were analyzed using a standardized spreadsheet to assess rehabilitation components and timelines. Descriptive statistics (median values, percentages, ranges) were calculated.

**Results:**

54 rehabilitation protocols were assessed, including 22 (41%) from ERAS-affiliated academic programs or associated physicians. Postoperative weight-bearing was addressed in 53/54 (98%) protocols, with 41 (77%) recommending toe-touch/touchdown/flat-foot/20-lb weightbearing. Median duration of restricted weight-bearing was 6 weeks (range, 2–8 weeks). Regarding range of motion restrictions, 37 (69%) protocols limited hip flexion to 90°, 42 (78%) advised against passive adduction past neutral, and 49 (91%) against active abduction. Bracing was recommended in 30 (56%) protocols, with 25 (83%) lacking brace specification and only 5 (17%) recommending an abduction brace. Return-to-sport timelines were included in 19 (35%) protocols, with a median 16 weeks (range 12–24). Functional testing was mentioned in 22 (41%) protocols, though tests and criteria varied.

**Conclusion:**

Substantial variability exists in the composition and timing of rehabilitation protocols following hip abductor tendon repair. A majority of protocols advised limited weight bearing for the first 6 weeks after surgery and avoidance of active hip abduction and passive hip adduction, however there was wide variation in the recommendations regarding brace use, strengthening progressions, the role of functional testing, and the timeline for return to activity and sport.

## Introduction

 The hip abductor complex, consisting of the gluteus medius and minimus muscles and their respective tendons, plays a crucial role in hip abduction and gait stability [1–3]. While acute injury is possible, tears of the hip abductor complex are often insidious in onset and degenerative in nature, most frequently occurring in women between the fourth and sixth decades of life [1, 4–7]. Nonoperative management is often first-line treatment, with modalities including activity modification and physical therapy to optimize joint biomechanics, as well as nonsteroidal anti-inflammatory medications and adjuncts such as corticosteroid injections [6]. Operative management is generally indicated for when symptoms are refractory to conservative measures, or for acute, traumatic tears or those associated with remarkable weakness or gait alteration [1]. Open and endoscopic techniques have been employed for hip abductor repair, with or without biologic augmentation, and have a profound impact on patient outcomes with improvements in pain, functional status, strength, and return to activity [4, 5, 7, 8]. Postoperative physical therapy is essential to optimize outcomes, as structured exercise programs can enhance strength and reduce pain within 4 to 12 months of rehabilitation [5].

Despite the effectiveness of post-operative physical therapy, the availability of consistent therapy protocols is lacking. Existing protocols can be highly variable, as many are sourced from individual expert-based recommendations rather than evidence-based trials or studies [4, 5]. In addition to the variability, many rehabilitation protocols have vague guidelines that lack detail, impacting both physical therapists and patients [4]. Prior investigations have found noteworthy variability in the timing and content of rehabilitation protocols after a variety of upper and lower extremity procedures; however, none have examined such protocols following hip abductor tendon repairs [7–14]. To address this gap, this study aimed to capture rehabilitation protocols that are easily available to physicians and physical therapists and through a descriptive analysis, demonstrate how these resources vary in both content and timing. Highlighting these inconsistencies may help to guide future evidence-based studies on appropriate timing and content of rehabilitation protocols to optimize clinical outcomes and return to activity. Additionally, identification of rehabilitation components with the most or least uniform initiation between protocols may guide future outcome-based studies to determine the impact of initiation date adherence on clinical outcomes.

The purpose of this study was to evaluate readily accessible U.S. online physical therapy protocols for open and endoscopic hip abductor repairs and to assess the variability in post-operative rehabilitation protocols. It was hypothesized that there would be pronounced heterogeneity among both content and timing of postoperative restrictions and rehabilitation exercises in publicly available rehabilitation protocols following hip abductor repairs.

## Methods

**Fig. 1:**
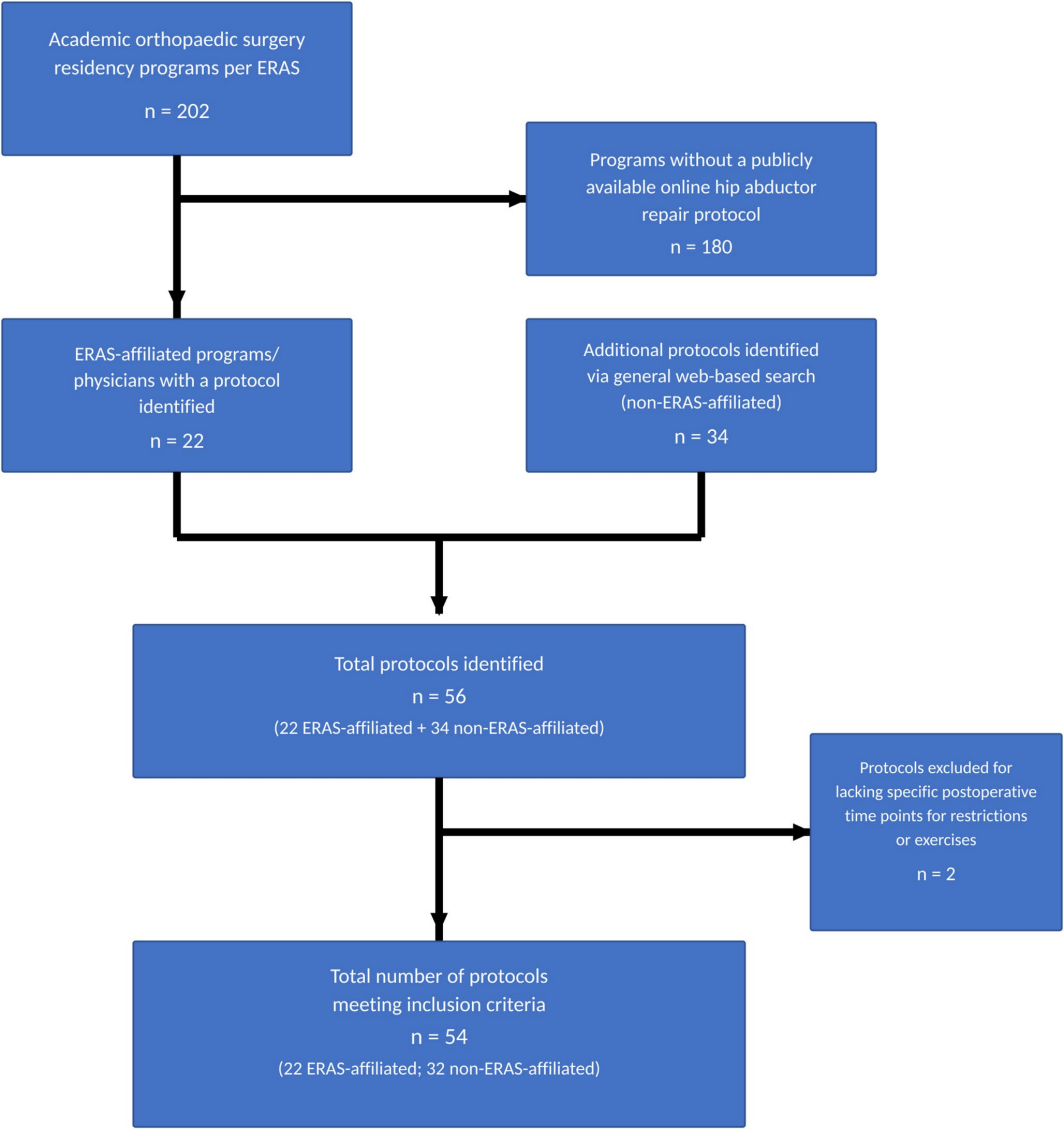
Flow diagram demonstrating identification, screening, eligibility, and inclusion of publicly available online hip abductor tendon repair rehabilitation protocols from U.S. orthopaedic surgery institutions

This study evaluated publicly available online physical therapy protocols from U.S.-based academic orthopaedic surgery programs found in the Electronic Residency Application Service (ERAS), which can be found officially on the Association of American Medical Colleges list of participating programs [15]. Online protocols were selected rather than manuscript-based or physical formats as they reflect rehabilitation guidance most readily accessible to patients and clinicians in real-world practice. In March of 2025, general web-based queries were performed (www.google.com) using “[ERAS program name] hip abductor repair rehabilitation protocol” and “[ERAS program name] gluteus repair rehabilitation protocol” to source publicly available protocols from ERAS programs and affiliated physicians. To increase the sample size and capture protocols most accessible to patients and physicians, an additional web-based query using the terms “hip abductor repair rehabilitation protocol” and “gluteus repair rehabilitation protocol” was performed. Exclusion criteria included protocols that were not U.S.-based or explicitly documented for rehabilitation following hip abductor repairs alongside a concomitant procedure, such as total hip arthroplasty. Restricting inclusion to U.S. based protocols minimized heterogeneity related to regional differences in healthcare systems, rehabilitation practices, and resource availability, while improving interpretability of protocol variability within a consistent training and practice environment. Additionally, protocols that failed to specify time points for postoperative restrictions or exercises were omitted from the study (Fig. 1).

Protocols were collected, consolidated and descriptively analyzed using a standardized spreadsheet (Microsoft Excel version 2506; Microsoft Corporation, Redmond, WA). Protocols were evaluated based on the inclusion and specific recommendations within key broad categories. These categories were defined as: adjunctive therapies, range of motion (ROM) restrictions, ROM/stretching exercises, strengthening, weightbearing, return to sport/activity, and functional testing. Given the large variability in nomenclature for weightbearing status, toe-touch, touchdown. flat-foot, and 20-lb weightbearing were all grouped together, as the clinical implications of these weight-bearing restrictions are largely similar. Non-weightbearing, 20–25% partial weightbearing (PWB), and 50% PWB and full weightbearing were listed as separate entities. The complete list of evaluated metrics can be found in Table 1. For every rehabilitation component present in a protocol within the categories, the recommended timeline of introduction and progression (where relevant) was recorded. Data were consolidated across all protocols and descriptive statistics were calculated to quantitatively evaluate the percentage of protocols including each rehabilitation component, including the median and range of recommended initiation times, when applicable. Overall methods were adapted from previously described methodology exploring variability of rehabilitation protocols following similar orthopaedic surgery procedures [7–14]. Institutional review board approval was not required as only publicly available data were used.

**Table 1:** Rehabilitation protocol categories and components

Adjunctive therapies	Scar massage, aqua therapy, bracing, NMES, cryotherapy, CPM, dry needling, blood flow restriction therapy, PRICE
Range of motion restrictions	No hip flexion > 90-degrees, no passive adduction, no active internal rotation, no passive external rotation, no active abduction, no hip extension past neutral
Range of motion and stretching exercises	Passive ROM, joint mobilization, glute/piriformis stretch, log roll, manual hip flexor stretching, IT-band stretching, quadruped rocking, stool rotations, active ROM, prone ER/IR, active-assisted ROM, prone lying, hamstring stretch, iliopsoas/rectus femoris stretch
Strengthening	Quadricep strengthening, core strengthening, hip isometrics, gait strengthening program, hip flexor strengthening, supine bridges, single-leg balance, leg press, lateral stepping, knee flexion/extension, hip extensions, step downs, hamstring strengthening, hip hiking, pelvic tilt
Weightbearing	TTWB/FFWB/20lb, 20–25% PWB, 50% PWB, NWB
Return to sport/activity	Stationary bike, proprioception training, running, elliptical, hip endurance, plyometrics, sports-specific drills, return to sports, swimming, return to activities of daily living
Functional testing	Hip outcome score, manual muscle testing, biodex test of quads/hamstrings, step down test, single-leg triple crossover hop, strength, single-leg hop, y-balance test

*NMES* neuromuscular electrical stimulation, *CPM* continuous passive motion, *PRICE* Protect Rest Ice Compression and Elevation, *ROM* range of motion, *IT* iliotibial, *ER* external rotation, *IR* internal rotation, *TTWB/FFWB/20lb* toe-touch/touchdown/flat-foot/20-lb weightbearing, *PWB* partial weightbearing, *NWB* non-weightbearing

## Results

A total of 54 rehabilitation protocols for hip abductor repairs were evaluated. Of these, 22 (41%) were sourced from ERAS-affiliated programs and physicians, while the remaining 32 were obtained from the websites of orthopaedic surgeons or practices not affiliated with an existing ERAS program. Of the included protocols, 36 (67%) protocols did not specifically state the utilized surgical technique (open versus endoscopic), while 4 (7%) of the protocols were directed for rehabilitation following open repair, 5 (9%) for endoscopic repair, and 9 (17%) for either open or endoscopic repair.

### Weightbearing

The status of initial weightbearing limitations was addressed in 53 of 54 protocols (98%) and categorized into four groups: non-weightbearing (NWB), toe-touch/touchdown/flat-foot/20-lb weightbearing (TTWB/TDWB/FFWB/20-lb), 20–25% partial weightbearing (PWB), and 50% PWB. Among these, 41 protocols (77%) recommended TTWB/FFWB/20-lb, 5 (9%) recommended 20–25% PWB, 5 (9%) recommended 50% PWB, and 2 (4%) prescribed NWB. None of the protocols that specifically commented on weightbearing recommended full weightbearing in the immediate postoperative period. The median duration of initial weight-bearing restrictions was 6 weeks (range of 2–8 weeks), with full weightbearing expected by eight weeks (range of 3–8 weeks). Nearly all protocols (52/54, 96%) recommended the use of an assistive device; of these, 30 (58%) advised crutches only, 16 (31%) suggested a walker as an alternative, and 3 (6%) did not specify the device.

### Range-of-motion restrictions

**Fig. 2:**
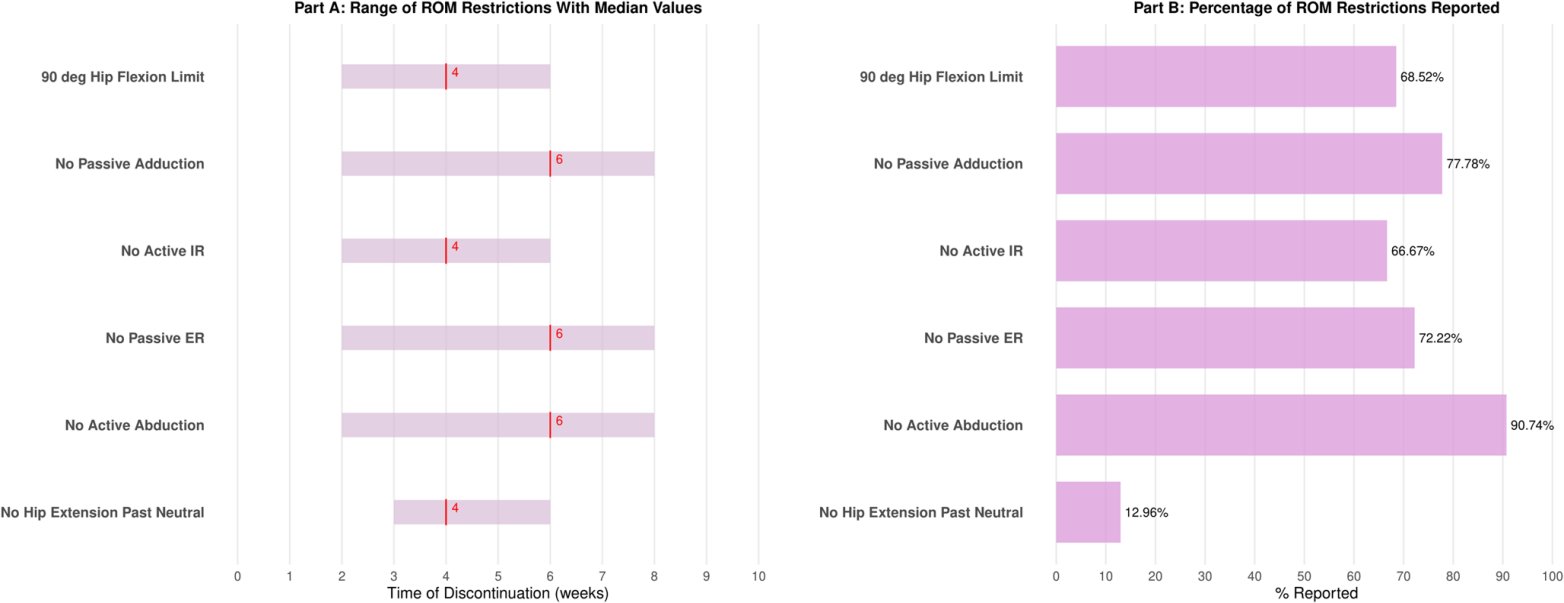
**a** median (numbered line) and range (shaded area) of ROM restriction discontinuation date, with **b** percentage of protocols containing each ROM restriction. *ER* external rotation, *IR* internal rotation, *ROM* range of motion

Protocols provided restrictions on hip flexion past 90 degrees, passive adduction, active internal rotation (IR), passive external rotation (ER), active abduction, and hip extension past neutral (Fig. 2). Of these six components, restrictions on active abduction, passive adduction, and passive ER were the most prevalent among the protocols. Out of 54 protocols, 49 (91%) limited active abduction for a median duration of 6 weeks (range, 2–8 weeks), 42 (78%) restricted passive adduction for a median of 6 weeks (range, 2–8 weeks), and 39 (72%) limited passive ER for a median of 6 weeks (range 2–8 weeks).

### Adjunctive therapy

**Fig. 3:**
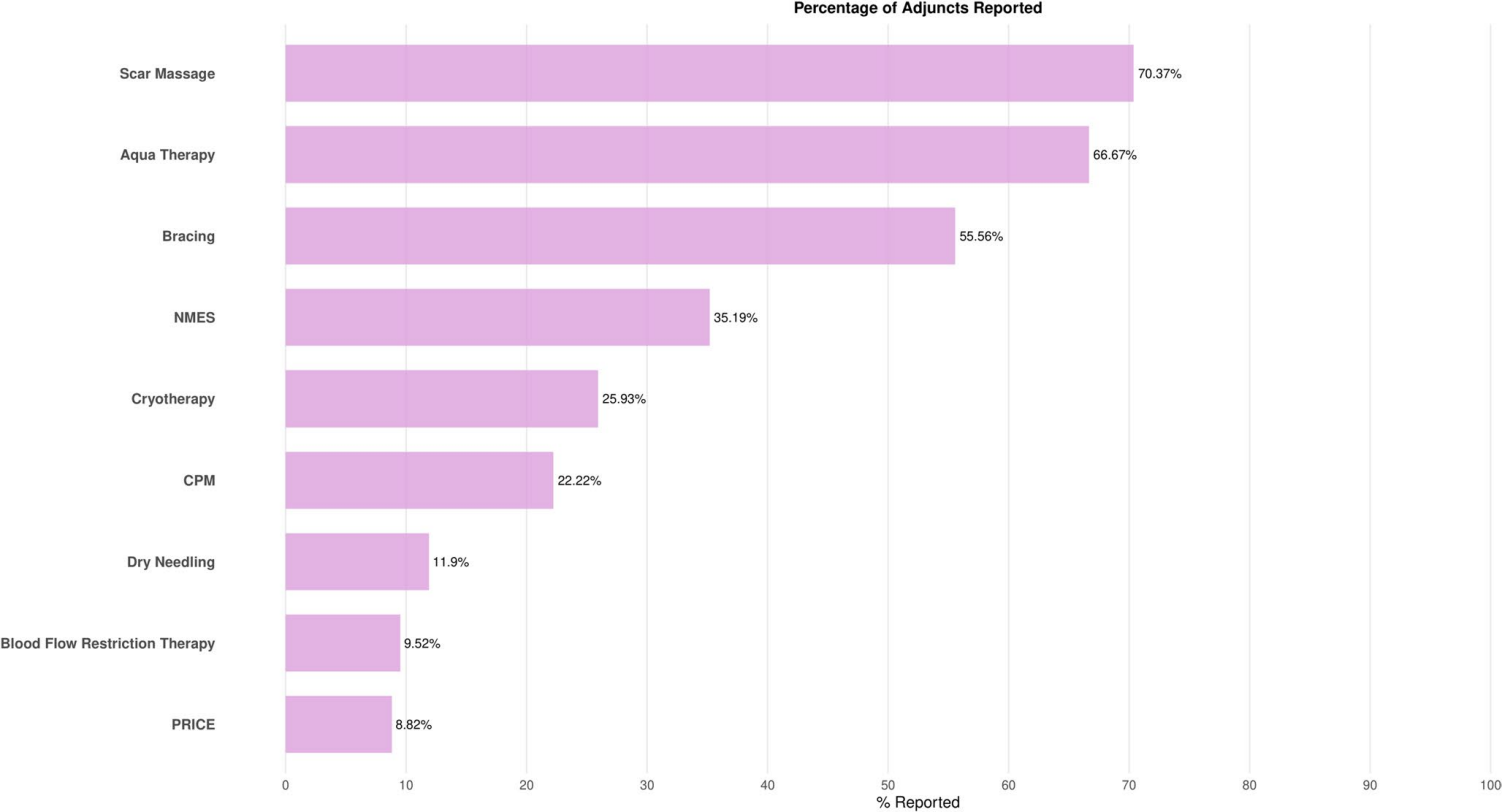
percentage of protocols including specific adjunctive therapies. *NMES* neuromuscular electrical stimulation, *CPM* continuous passive motion, *PRICE* Protect Rest Ice Compression and Elevation

There were 30 (56%) protocols that recommended the use of a brace postoperatively. Of these 30, only 5 (17%) protocols specified the type of brace that was to be used, which was a hip abduction brace; the remaining 25 did not specify. Nine types of postoperative adjunctive therapies were reported across all protocols (Fig. 3). Scar massage, aqua therapy, and bracing were most frequently recommended and were reported in 70%, 67%, and 56% of protocols, respectively.

### Range of motion and stretching

**Fig. 4:**
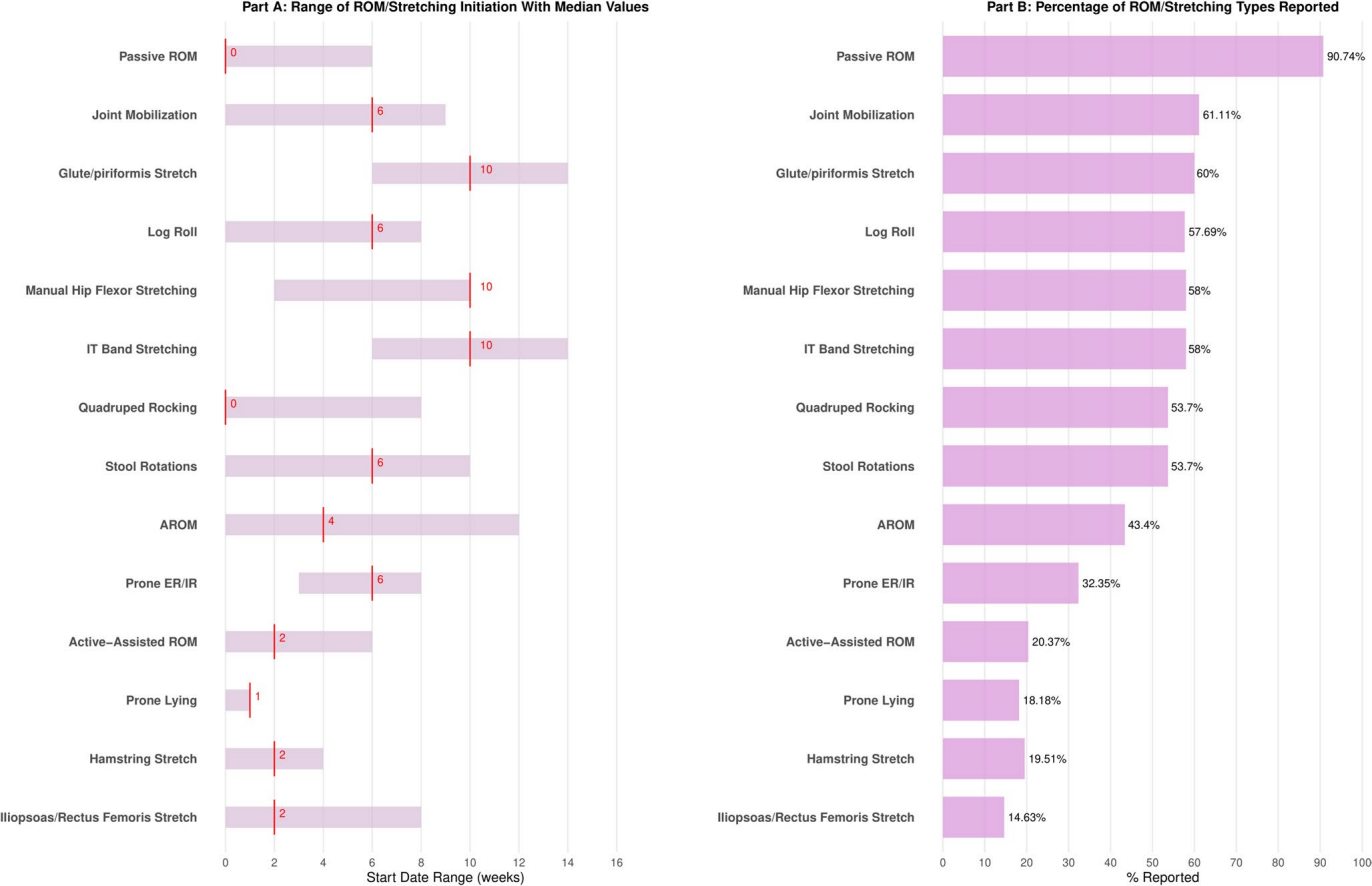
**a** median (numbered line) and range (shaded area) of ROM/Stretching exercise initiation, with **b** percentage of protocols containing each specific exercise. *AROM* active range of motion, *ER* external rotation, *IR* internal rotation, *ROM* range of motion, *IT* iliotibial

49 (91%) protocols recommended the usage of passive range of motion (PROM) exercises with a median initiation time of 0 weeks postoperatively (Fig. 4). Other commonly included activities were joint mobilizations (61%), glute/piriformis stretches (60%), and log rolling (58%). Active range of motion, which was reported in 43% of protocols, had the widest recorded range of recommended start times, spanning from zero to 12 weeks postoperatively. Fewer protocols incorporated activities such as prone lying (18%), hamstring stretches (20%), and iliopsoas/rectus femoris stretching (15%).

### Strengthening

**Fig. 5:**
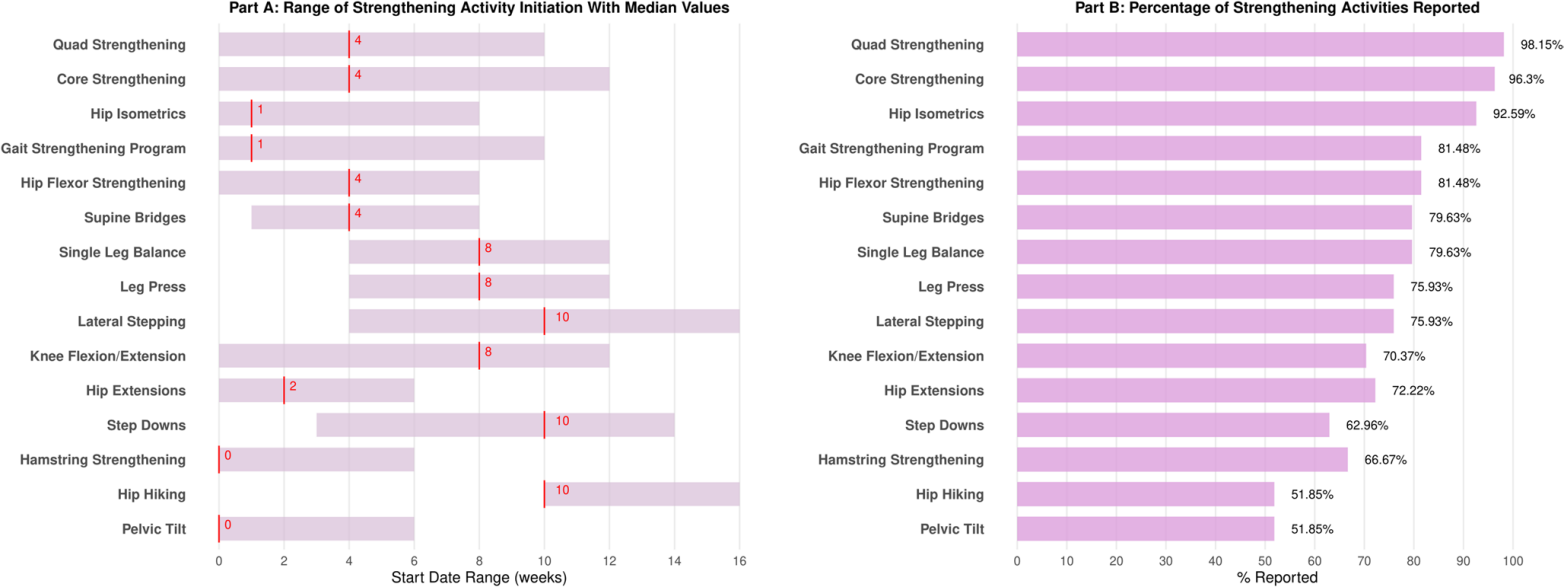
**a** median (numbered line) and range (shaded area) of strengthening exercise start date, with **b** percentage of protocols containing each specific exercise

57 distinct exercises were identified across all protocols. The 15 most frequently reported exercises are listed in Fig. 5, with all of them appearing in more than 50% of the 54 collected protocols. Quadriceps strengthening was the most included activity, recommended in 53 protocols (98%), followed by core strengthening (96%) and hip isometric exercises (93%). Hip hiking had the latest recommended initiation, beginning as late as 10 weeks postoperatively, and was one of only two exercises, along with lateral stepping, with a suggested start time extending to 16 weeks. Knee flexion/extension, core strengthening, and lateral stepping showed the greatest variability in initiation timing, with a 12-week range across protocols.

### Return to activity and functional testing

**Fig. 6:**
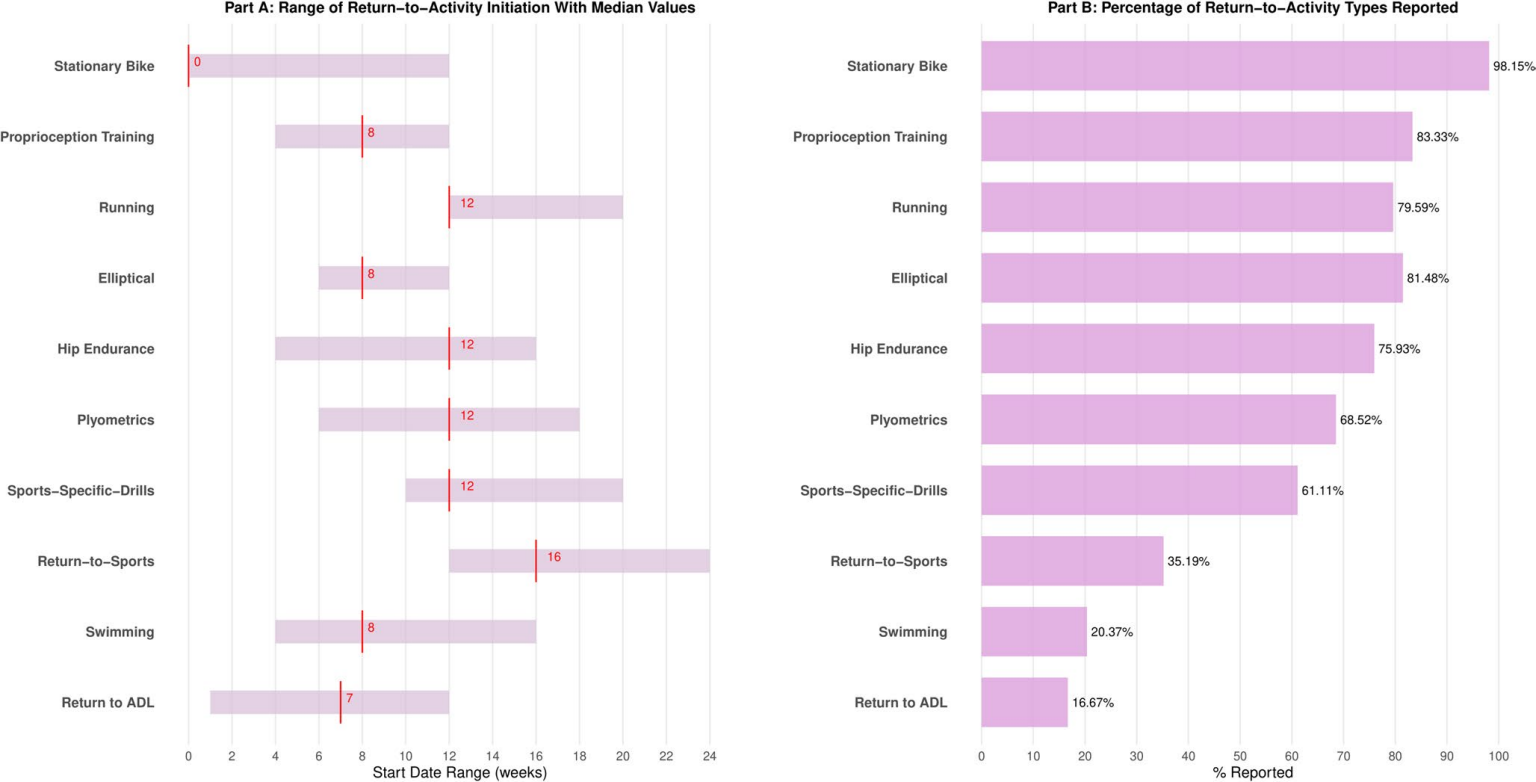
**a** median (numbered line) and range (shaded area) of return to activity/sport start date, with **b** percentage of protocols containing each specific activity or sport. *ADL* activities of daily living

Ten activities were reported across protocols, seven of which were included in over 50% of the collected protocols (Fig. 6). The most frequently reported activity was stationary biking (98%), followed by proprioception training (83%), running (80%), elliptical (81%), and hip endurance exercises (76%). Stationary biking had the earliest median start at 0 weeks postoperatively. Regarding return to sport, 33 (61%) protocols incorporated sport-specific drills at a median initiation timeline of 12 weeks post-operation. However, only 19 (35%) protocols explicitly mentioned a return-to-sport goal or phase for hip abductor repair rehabilitation, with a median start of 16 weeks post-operation (range 12–24 weeks).

22 protocols (41%) included functional testing in any capacity. Among these, 12 (55%) used it as a criterion for discharge, 8 (36%) required it prior to return to sport, and 5 (23%) employed it to determine progression to the next phase of therapy. There was great variability in which functional tests were utilized among the protocols, but the most frequently utilized were the patient-reported Hip Outcome Score (20%), Manual Muscle Testing (19%), and the Biodex test of the quadriceps and hamstrings (19%). For Manual Muscle Testing, a score 90% or greater of the nonoperative leg was considered acceptable, while for the Biodex test, a peak torque 85% or greater of the nonoperative leg was the desired benchmark.

## Discussion

The main finding from the present study was the identification that substantial variability exists in both content and timing of publicly available post-operative rehabilitation protocols for patients following open and endoscopic hip abductor tendon repairs. Furthermore, these findings demonstrate the lack of publicized protocols, with only a small minority (11%, 22/202) of accredited residency-training academic orthopaedic surgery institutions or their affiliated surgeons disseminating standardized rehabilitation protocols. Ebert et al. performed a recent systematic review of post-operative rehabilitation protocols described in published clinical studies and similarly found no evidence-based rehabilitation studies after hip abductor tendon repairs and furthermore found variability amongst documented rehabilitation protocols. In response, the authors proposed a 7-phase rehabilitation protocol as a potential foundation for future reference and investigation [4]. However, the protocols described in the studies reviewed by Ebert et al. were typically presented as postoperative recommendations or a limited overview of the rehabilitation course, often lacking specific exercise prescriptions, ROM restrictions, or clearly defined progression timelines [4].

In contrast, the present study descriptively evaluates rehabilitation protocols that are directly accessible to surgeons, physical therapists, and patients and demonstrates that even among these commonly referenced resources, there remains considerable heterogeneity in both rehabilitation content and timing. While variation is often necessary in the setting of concomitant procedures (e.g., hip abductor tear repair performed alongside hip arthroscopy or arthroplasty), intra-operative findings (such as notable fatty atrophy requiring augmentation), or repair techniques, prior orthopaedic research has demonstrated that standardization of protocols and clinical pathways can enhance outcomes, improve efficiency, and reduce costs, emphasizing the utility of standardized, evidence-based protocols [4, 16–21]. By highlighting where rehabilitation protocols for hip abductor repairs are both most variable and most consistent, the findings can serve as framework for subsequent evidence-based research on how standardization influences clinical outcomes and patient satisfaction, if at all.

Across the seven evaluative categories of this study, there was heterogeneity between protocols; however, several rehabilitation domains demonstrated relative consistency. Most protocols recommended ROM restrictions regarding passive adduction and active abduction of the hip for 6 weeks post-operatively, as well as the promotion of limited WB through non-WB as well as a variation of TTWB/TDWB/FFWB/20-lb WB. These findings suggest an importance to reduce premature stress to the repair in the early post-operative period. In contrast, considerable variability was noted in recommendations regarding brace utilization, strengthening progression, functional testing, and return-to-sport criteria. The variability in brace utilization likely reflects ongoing uncertainty regarding its cost-effectiveness and the extent to which patient education alone can adequately prevent compromising movements during the early healing phase (4, 28). Strengthening exercises demonstrated the greatest heterogeneity, likely due to the broad range of exercises available to target overlapping muscle groups and differences in how clinicians balance restoration of strength with protection of the repair. Functional testing was inconsistently incorporated and varied considerably in methodology, and with only 12 and five protocols using functional testing as criterion for phase advancement and discharge from rehabilitation respectively, there is potential opportunity to improve patient outcomes with more widespread incorporation. Finally, variability in return-to-sport recommendations may be attributable to differences in patient goals and demographics, as many individuals undergoing hip abductor repair are middle-aged or older patients seeking restoration of daily function rather than return to competitive athletics. However, with only 17% of protocols highlighting a return to ADLs as a benchmark, an important milestone for older populations may be overlooked. Collectively, these findings suggest broad agreement regarding protection of the repair during early healing, but less consensus regarding the optimal progression of later-stage rehabilitation.

While all but one published protocol specifically commented on postoperative weightbearing restrictions, there was variability among initial weightbearing restrictions as well as time to return to full weightbearing. Most protocols (77%) recommended TTWB/TDWB/FFWB/20-lb, while others advised 20–25% PWB (9%), 50% PWB (9%), or NWB (4%). The median duration of initial PWB was six weeks, with full WB typically permitted by eight weeks. Notably, similar weightbearing restrictions were described using varying terminology (toe-touch, touchdown, flat-foot, 20-lb), which may further contribute to perceived variability though they presumably represent a similar restriction. While limited data exists on the biologic healing timeline of hip abductor tendon repairs, extrapolated evidence from rotator cuff healing models suggest macroscopic healing occurs at approximately eight weeks, however histologically this may range from 12 to 15 weeks [22]. As weightbearing requires considerate generation of abductor force to counteract body weight and maintain gait and balance, premature weightbearing may stress the repair prior to full healing and compromise overall repair integrity. Prior studies have also suggested varying time to full weightbearing based on tear size, presuming that larger tears may require more conservative timelines [23]. Given these restrictions, aquatherapy/hydrotherapy, which was discussed in 67% of protocols, may be a useful adjunct, enabling earlier exercise while protecting the repair following wound healing [4, 24, 25].

Similarly, excessive ROM may place undue stress on the repair construct. Nearly all protocols included ROM restrictions in early rehabilitation phases, as excessive ROM has the possibility of directly stressing the repair as well as compressing the abductor complex due to the anatomic relationship of the iliotibial band [24–27]. The protocols in the current study most commonly restricted active abduction (80%) and passive adduction (78%) for a median 6 weeks (range 2–8 weeks). In a similar manner, bracing was mentioned in 56% of the protocols; however, only 17% specified use of a hip abduction brace. While the goal of bracing is to limit compromising movements, the lack of uniformity on this matter likely reflects debate about cost-effectiveness and whether patient education can offer comparable benefit [4, 28].

Strengthening exercises by far had the largest amount of variability across included protocols in the current study, which may be due to the wide range of available exercises targeting overlapping muscle groups. Overall, the goal of strengthening exercises in rehabilitation after hip abductor repair is to recover full strength progressively to > 95% of the contralateral side, while maintaining phase-specific restraints and avoiding re-injury [4]. To that matter, it is reasonable that simple exercises that avoid excessive hip range of motion or strength in hip abduction (e.g., hip isometrics, pelvic tilts, hamstring strengthening) have earlier median start dates, while compound movements and those incorporating hip abduction were not recommended to begin until later median start dates (e.g., lateral stepping, step downs, hip hiking).

Although prior clinical outcome studies have demonstrated successful return to activity after surgical repair, with a recent systematic review finding weighted satisfaction rate of 91% with return to activity most commonly at 3–8 months, return-to-sport guidance and functional testing were inconsistently addressed in the current study, with only 41% of protocols including functional testing in any capacity [29]. However, 61% of protocols did recommend sport-specific drills to be incorporated (median initiation at 12 weeks). More basic activities were introduced progressively throughout rehabilitation, with stationary biking being the earliest activity suggested at a median start at week 0 postoperatively. More gradual increasing impact activities were generally introduced at the 12-week mark. The lack of strict return-to-sport criteria may reflect the typical patient demographic (middle-aged women), for whom high-impact athletic performance is less frequently prioritized compared to those recovering from anterior cruciate ligament reconstruction, for example [6]. Given this demographic, a focus on a return to activities of daily living (ADLs) may be of greater utility to both physicians and patients, yet only 17% of protocols explicitly highlighted this milestone. Nevertheless, it remains important for therapists to minimize deficits in recognizable areas that may preclude patients from successful return to activity, whether related to physical function, dynamic biomechanics or mental preparedness [4, 28].

Of note, endoscopic repairs of gluteus minimus and medius tears have become more prevalent since 2007, with studies demonstrating reduced complication rates while also maintaining similar efficacy and recovery outcomes to an open repair technique [30–33]. As mentioned in the results section, 36 (67%) of the 54 collected protocols did not specify the surgical technique used for hip abductor repair. This may reflect an understanding from those composing rehabilitation protocols that from a post-operative perspective, there is no notable difference between open and endoscopic repairs. Between the two techniques, this study collected only four (7%) and five (9%) protocols that specified rehabilitation for open and endoscopic repairs, respectively, and given these smaller percentages, alongside the aforementioned similar clinical outcomes, separating protocols based on technique would not have made meaningful impact on the results of this study.

### Limitations

This study had several notable limitations. First, as published protocols were accessed via general web-based query, it is possible that additional protocols were not captured, including those published exclusively within scientific manuscripts or disseminated through institutional internal platforms. As such, protocols that are only accessible to surgeons, physical therapists, or patients through direct communication were not included. Second, the goal of the current study was to evaluate rehabilitation protocols specific to hip abductor repair alone; there may be varying recommendations in those performed alongside other procedures such as total hip arthroplasty or hip arthroscopy. Furthermore, while considerable variability was observed across protocols, there is currently no established quantitative benchmark for acceptable variability in postoperative rehabilitation, which limits objective comparison between protocols. Finally, this study did not assess the quality or clinical effectiveness of individual rehabilitation protocols, nor did it correlate protocol characteristics with patient-reported or functional outcomes. Additionally, ERAS affiliation was used solely as a systematic method to identify publicly available protocols associated with academic orthopaedic surgery program, which do not necessarily reflect institutional rehabilitation standards. The intent of this investigation was to characterize the variability of rehabilitation guidance readily accessible to clinicians and patients, rather than to determine the clinical superiority of any specific rehabilitation strategy.

## Conclusion

Substantial variability exists in the composition and timing of rehabilitation protocols following hip abductor tendon repair. A majority of protocols advised limited weight bearing for the first 6 weeks after surgery and avoidance of active hip abduction and passive hip adduction, however there was wide variation in the recommendations regarding brace use, strengthening progressions, the role of functional testing, and the timeline for return to activity and sport (Fig. 1).

## Data Availability

All rehabilitation protocols included in this study were archived in a publicly accessible repository to promote transparency and reproducibility. The dataset contains the full list of source URLs and institutional affiliations. 10.5281/zenodo.18476126.
